# Dipyridamole-induced STEMI: case report and review of the literature

**DOI:** 10.21542/gcsp.2023.23

**Published:** 2023-08-01

**Authors:** Hussam Al Hennawi, Sunita Lakhani, Shayan Iqbal Khan, Sarin Atam, Usama Sadiq, Joseph Rigotti, Abhinav Nair

**Affiliations:** 1 Department of Internal Medicine, Jefferson Abington Hospital, Abington, PA, USA; 2 Department of Cardiology, Thomas Jefferson University Hospitals, Philadelphia, PA, USA; 3 Department of Cardiology, Jefferson Abington Hospital, Abington, PA, USA

## Abstract

Dipyridamole nuclear myocardial perfusion imaging is a safe and useful modality for assessing myocardial ischemia. It is the modality of choice for cardiac risk stratification in patients who are unable to exercise. Intravenous dipyridamole causes coronary vasodilation and may result in heterogeneity of coronary blood flow in significant coronary artery disease. Ischemic electrocardiographic changes following pharmacological stress testing are less likely to occur compared with exercise stress tests. Ischemia is more likely to be present in the form of ST depression, with ST-segment elevation being exceedingly rare. We present the case of a 73-year-old patient who developed ST-segment elevation myocardial infarction following pharmacologic stress testing.

## Introduction

Nuclear myocardial perfusion imaging (MPI) is a safe and effective modality for detecting myocardial ischemia in patients who are unable to undergo exercise stress testing^1^. Dipyridamole and regadenosine are as sensitive and specific for detecting myocardial ischemia as dobutamine, adenosine, and exercise stress testing^1,2^. In fact, when compared with exercise myocardial perfusion testing, dipyridamole nuclear testing has similar sensitivity (79%) and higher specificity (95% vs. 92%) for the detection of coronary artery disease (CAD)^3^.

Ischemic electrocardiographic (EKG) changes during dipyridamole stress testing may manifest as ST depression and possible angina^4^. ST-elevation myocardial infarction (STEMI) is an exceedingly rare complication^4^. Potential mechanisms of myocardial injury include vasodilator-induced myocardial ischemia (coronary steal), coronary vasospasm, or plaque rupture^5^. We report the case of a 73-year-old male patient who developed STEMI a few hours after dipyridamole MPI with evident severe stenosis on subsequent coronary angiography. To the best of our knowledge, this is the first report of a delayed complication following dipyridamole stress testing.

## Case presentation

A 73-year-old male with poorly controlled insulin-dependent diabetes mellitus, hypertension, CAD with remote coronary artery bypass graft (CABG) 30 years prior, and severe peripheral artery disease (PAD) presented to an outside hospital for surgical revascularization of the right lower extremity with possible amputation.

**Figure 1.**
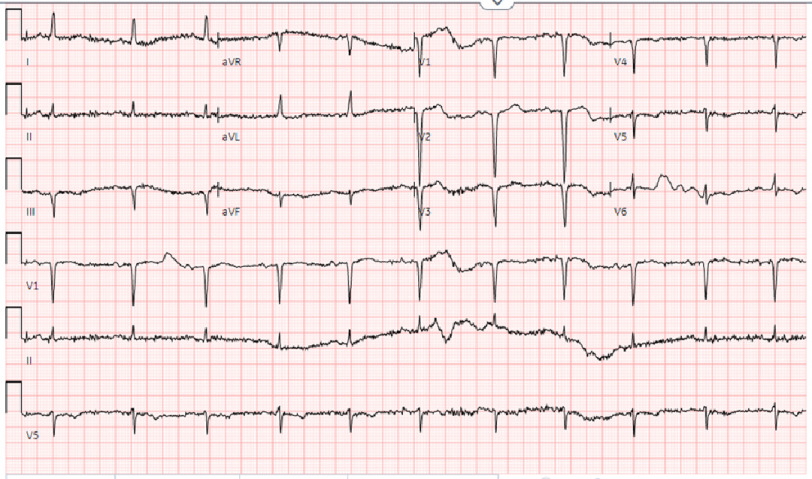
Electrocardiogram before pharmacological stress test showing sinus rhythm anterior infarct and non-specific T-wave abnormalities.

On arrival, the patient was hemodynamically stable and denied any anginal symptoms. The patient was taking aspirin (81 mg), atorvastatin (20 mg), and insulin. He was being treated for cellulitis and osteomyelitis of the right leg wound prior to arrival. An echocardiogram performed 2 weeks prior showed an ejection fraction (EF) of 45% with inferior hypokinesis. Given his medical comorbidities and no recent ischemic evaluations, he was scheduled for preoperative pharmacologic stress testing to better characterize the burden of ischemia. Pharmacological stress testing with dipyridamole was administered intravenously at the rate of 0.56 mg/kg over 4 min. Baseline electrocardiogram (EKG) showed sinus rhythm with nonspecific T-wave changes (Figure 1).

**Figure 2.**
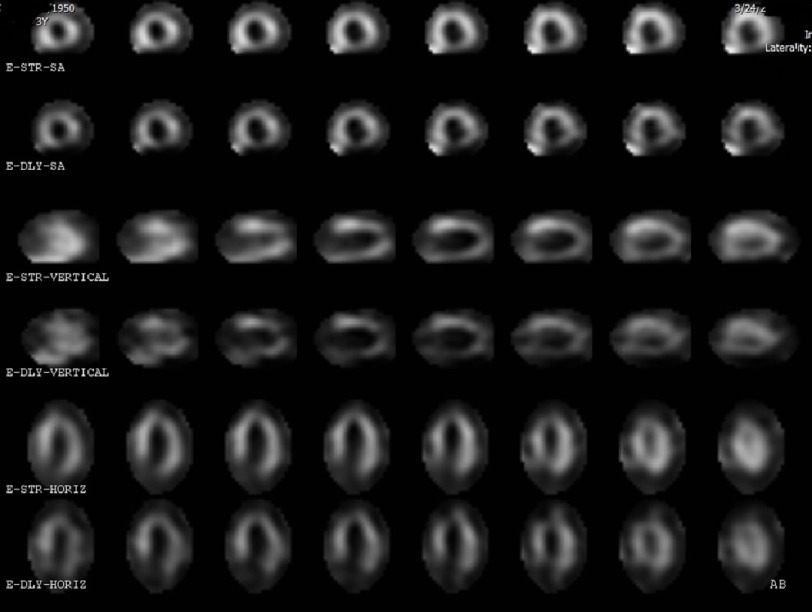
Left ventricular myocardial perfusion imaging showing abnormal perfusion with no notable left ventricular perfusion ischemia.

**Figure 3.**
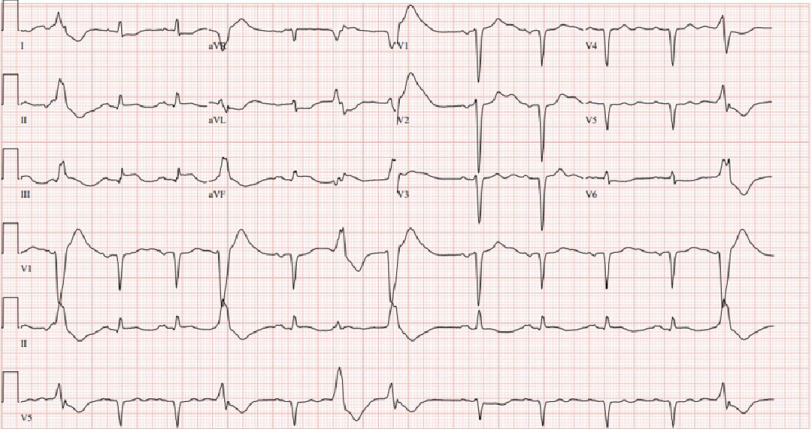
Electrocardiogram post-arrest showing wide QRS rhythm with occasional premature ventricular complexes and ST-elevation consistent with inferior infarct and reciprocal ST depression.

**Figure 4.**
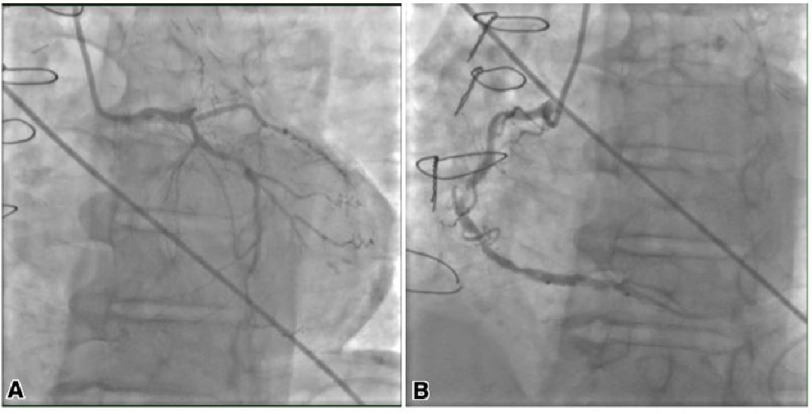
Coronary angiography showing mid-left main coronary artery with an estimated 30% stenosis (A). Left anterior descending with 100% lesion. Mid-right coronary artery with 90% stenosis (culprit lesion) and distal right coronary artery with 50% stenosis (B).

The patient experienced no symptoms during the stress test and had a normal hemodynamic response to stress testing. No significant ECG changes were observed during the stress testing. MPI was completed uneventfully, with no perfusion defects. However, left ventricular systolic function was moderately decreased with global hypokinesis along with anteroseptal and apical anterior akinesis (Figure 2). At this time, he was recommended to undergo surgery without additional cardiac diagnostics/ interventions at moderate to high risk.

Two hours after the stress test, the patient became unresponsive and experienced cardiac arrest. The initial rhythm was ventricular fibrillation (VF). ACLS was initiated, the patient was intubated, defibrillation was performed three times, and a return of spontaneous rhythm was achieved in 6 min. Post-arrest EKG showed a new inferior ST elevation with reciprocal ST depression in the anteroseptal leads (Figure 3). The patient was immediately taken to the catheterization laboratory for further evaluation.

Coronary angiography revealed severe multivessel CAD, 30% stenosis of the mid-left main artery, 50% stenosis of the distal right coronary artery (RCA), and 90% stenosis of the mid-RCA (Figure 4). Percutaneous coronary intervention (PCI) was performed with successful delivery of a bare metal stent (BMS) to the mid-RCA. The patient was noted to have recurrent myoclonic jerking during catheterization, and was deemed to have a grave neurologic prognosis. He was managed in the cardiac care unit; however, in the absence of neurological improvement, was extubated palliatively as per family wishes.

## Discussion

Dipyridamole-induced STEMI is an exceedingly rare complication reported only in a few previous scientific literature^6–11^. A review conducted by Lette et al. reported an incidence of 0.95 deaths and 1.76 nonfatal infarctions per 10,000 patients who received dipyridamole for stress testing^2^. The safety of dipyridamole and other vasodilatory pharmacological agents is due to the production of hyperemia with no associated significant increase in myocardial oxygen demand^12^.

**Table 1:** Overview of dipyridamole-induced ST-elevation cases from the literature.

Author	Reference	Age and Sex	Post-stress EKG	Mechanism	Initial management	Catheterization report	Eventual Management
Al Hennawi et al.	CR	73, M	Unremarkable	Plaque rupture	None (late presentation)	30% mid-LMCA stenosis, 100% LAD stenosis. 90% mid-RCA stenosis (culprit lesion) and 50% distal RCA stenosis	PCI to RCA
Hansen et al.	6	47, M	ST-segment elevation	Vasodilatory-induced coronary steal	Aminophylline, SL NTG	Total occlusions of RAD, LAD, occluded distal circumflex and 60% stenosis of the first oblique marginal	CABG
Mutlu et al.	7	44, F	ST-segment elevation	Vasodilatory-induced coronary steal	Aminophylline, SL NTG	100% RCA, 70% LAD, 60% of the 2nd diagonal branch, 70% the 3rd diagonal branch, and 70% occlusion of the proximal circumflex artery	CABG
Safi et al.	8	56, M	ST-segment elevation	Vasodilatory-induced coronary steal	None	LCX total occlusion and proximal 40–50% stenosis of first obtuse marginal branch	Medical management
Kwai et al.	9	64, M	ST-segment elevation	Vasodilatory-induced coronary steal	SL nifedipine, nitropaste, NTG, and morphine	Occlusion of all bypass grafts, the native LAD, RCA, and a 90% stenosis of the LMCA	Streptokinase
Li et al.	10	67, F	ST depression progressing to ST-segment elevation	Unstable plaque rupture	Aminophylline	Subtotal distal RCA occlusion	PCI to RCA using a drug- coated balloon
Castro et al.	11	73, F	ST-segment elevation	Coronary vasospasm	SL NTG	Unremarkable	Medical management

**Notes.**

CR current SL sublingual NTG nitroglycerin ASA aspirin RCA right coronary artery LAD left anterior descending artery LCX left circumflex artery LMCA left main coronary artery PCI percutaneous coronary intervention

Dipyridamole-induced ST-segment elevations have been observed in patients without coronary lesions at the end of test procedures and after aminophylline administration, which is attributed to the sudden termination of vasodilatory stimulation^11–14^. Other mechanisms, including coronary vasospasm and coronary steal phenomenon were contributory^11,15^. Our patient developed cardiac arrest approximately 5 h after undergoing dipyridamole stress testing. Notably, he was asymptomatic during and shortly after the nuclear test, and there was no concern of any hemodynamic alterations that may have explained any impending acute coronary vessel disease (Table 1).

It is unlikely that pharmacological vasodilation directly contributed to ischemia, especially given the temporal delay. Delayed STEMI may have been related to unstable plaque rupture in the setting of extensive atherosclerotic burden. It is feasible that coronary hyperemia contributed to this phenomenon.

It also bears mentioning that STEMI might only be coincident with prior stress testing and that pharmacological testing was not actually causative. To our knowledge, this is the first case describing STEMI hours after dipyridamole stress testing in comparison to previously reported cases that described such complications during the period of pharmacological stress. This case adds to the literature on the importance of continuous cardiac monitoring in patients following pharmacological stress tests.

## What we have learned?

• Dipyridamole myocardial perfusion imaging is relatively safe.
• Coronary ischemia is one of the complications during nuclear stress testing.
• Myocardial ischemia may stem from vasodilator-induced myocardial injury, coronary vasospasm, or spontaneous plaque rupture.

## References

[1] Zamorano J, Duque A, Baquero M, Moreno R, Almeria C, Rodrigo JL (2002). Stress echocardiography in the pre-operative evaluation of patients undergoing major vascular surgery [in Spanish]. Rev Esp Cardiol.

[2] Lette J, Tatum JL, Fraser S, Miller D, Waters DD, Heller G, Stanton EB (1995). Safety of dipyridamole testing in 73.806 patients. J Nucl Cardiol.

[3] Leppo JA (1989). Dipyridamole-thallium imaging: the lazy man’s stress test. J Nucl Med.

[4] Henzlova MJ, Duvall WL, Einstein AJ, Travin MI, Verberne HJ (2016). ASNC imaging guidelines for SPECT nuclear cardiology procedures: stress, protocols, and tracers. J Nucl Cardiol.

[5] Li KFC, Ho HH, Yew MS (2019). A case report of dipyridamole stress-induced ST depression progressing to ST-elevation myocardial infarction despite intravenous aminophylline: steal, spasm, or something else?. Eur Heart J Case Rep.

[6] Hansen CL, Williams E (1998). Severe transmural myocardial ischemia after dipyridamole administration implicating coronary steal. Clin Cardiol.

[7] Mutlu H, Leppo J (2007). Coronary steal and ST elevation during dipyridamole stress testing leading to coronary artery bypass grafting. J Nucl Cardiol.

[8] Safi AM, Pillai N, Rachko M, Chaudhry K, Stein RA (2001). Dipyridamole-induced ST-segment elevation indicative of transmural myocardial ischemia–a case report. Angiology.

[9] Kwai AH, Jacobson AF, McIntyre KM, Williams WH, Tow DE (1990). Persistent chest pain following oral dipyridamole for thallium 201 myocardial imaging. Eur J Nucl Med.

[10] Li KFC, Ho HH, Yew MS (2019). A case report of dipyridamole stress-induced ST depression progressing to ST-elevation myocardial infarction despite intravenous aminophylline: steal, spasm, or something else?. Eur Heart J Case Rep.

[11] Díaz-Castro O, Fernández-López J, Campos L, Calvo F, Mantilla R, Goicolea J (2004). Elevación del segmento ST durante una tomografía computarizada por emisión de fotones simples con dipiridamol en ausencia de lesiones coronarias [ST segment elevation during dipyridamole stress testing in a patient without coronary lesions]. Rev Esp Cardiol.

[12] West J, Bellet S, Manzoli U, Miiller 0 (1962). Effects of persantin (RA8), a new coronary vasodilator, on coronary blood flow and cardiac dynamics in the dog. Circ Kes.

[13] Wendt V, Sunderrneyer J, den Bakker P (1962). Bing R: The relationship between coronary blood flow, myocardial oxygen consumption and cardiac work as influenced by persantin. Am J Ccirdiol.

[14] Picano E, Lattanzi F, Masini M, Distante A, L’ Abbate A (1988). Aminophylline termination of dipyridamole stress as a trigger of coronary vasospasm in variant angina. Am J Cardiol.

[15] Weinmann P, le Gudulec D, Moretti JL (1994). Coronary spasm induced by dipyridamole during dipyridamole scintigraphy. Int J Cardiol.

